# Supplementary material for: Transformation of temporal sequences in the zebra finch auditory system
Source: eLife. 2016 Nov 29;5:e18205. doi: 10.7554/eLife.18205 (PMC5161447; doi:10.7554/eLife.18205)
Supplement: Figure 6—source data 1. — The success of operant training was determined on the basis of the d-prime score. When d’ is greater than 1, the bird was deemed successful in learning the task. In this table, the number of birds that succeeded in operant training for click sequence discrimination (d’ > 1) out of the total number of birds is shown. For example, 8 out of 10 birds succeeded in two-stage training to discriminate sequence 9 and 2. DOI: http://dx.doi.org/10.7554/eLife.18205.019 [file elife-18205-fig6-data1.docx]

| Training sequence | | Seq. 9 vs 2 | | Seq. 1 vs 2 | |
| --- | --- | --- | --- | --- | --- |
| Timescale | | 1x | 2x | 1x | 2x |
| Training Method | Single-stage | - | - | 8/18 | 0/11 |
|  | Two-stage | 8/10 | - | 8/10 | 0/4 |
